# Fat Graft, Laser CO₂ and Platelet-Rich-Plasma Synergy in Scars Treatment

**Published:** 2013-12-25

**Authors:** AC Nita, OA Orzan, M Filipescu, D Jianu

**Affiliations:** * Dermatology Department of “ProEstetica” Medical Center, Bucharest, Romania; ** Dermatology Department, Elias University Emergency Hospital, Bucharest, Romania; *** “Carol Davila” University of Medicine and Pharmacy, Bucharest, Romania; **** Plastic Surgery Department of “ProEstetica” Medical Center, Bucharest, Romania

**Keywords:** scar, fat graft, platelet-rich plasma, preadipocyte

## Abstract

Abstract

Rationale: Many treatments have been proposed for cosmetic or functional improvement of scars. It is known that fat grafts and laser treatment can have beneficial effects on the remodeling of scar tissue, and platelet-rich plasma (PRP) can be effective during the wound-healing process. We hypothesized that laser and PRP can enhance fat graft survival and the combination would be effective in improving scars appearance.

Objective: The purpose of this study was to evaluate the efficacy of these combinations in the treatment of atrophic and contractile scars.

Methods and Results: From 2008-2013, we treated with this combination 64 patients affected by atrophic and contractile scars involving different body parts. At 6 months the patients’ overall satisfaction rate was excellent for over 50% of the patients.

Discussion: The association of an ablative laser CO2 with PRP and autologous fat graft seems to be a promising and effective therapeutic approach for atrophic and contractile scars.

Abbreviations: PRP platelet-rich plasma, OTI orotracheal intubation, HLLT high level laser therapy, LLLT low level laser therapy

## Introduction

All wounds leave scars, unless they are very small or superficial; over 100 million people acquire postsurgical scars each year in the developed world alone [**1**]. Skin scars have a unique impact on patients’ lives; so many treatments have been proposed for cosmetic and functional improvement of scars.

Regenerative medicine is an emerging and rapidly evolving field of research and therapies, thanks to the new discovery on stem cells. The discovery of preadipocytes, their mesenchymal origin, and their role as pluripotent stem cells have been used to maintain graft tissue [**2**,**3**]. So, fat grafting, a well-established technique in surgery, became an important tool in regenerative medicine due to the preadipocytes’ capability to differentiate and its role in collagen synthesis and angiogenesis [**4**]. Nevertheless, the major problem remains the ability to maintain fat graft survival and to produce more preadipocytes.

PRP is plasma with a higher concentration of platelets, >300 – 350 x 10³ platelets/μL, an increase of up to 3-5 times than normally found [**5**]. The α-granules of the platelets release growth factors in response to platelet activation, and stimulate cell proliferation and cell differentiation for tissue regeneration. These growth factors have an important role in the regulation and proliferation of mesenchymal cells, including fibroblasts and have been shown to reduce healing time and improve the likelihood of complete wound healing – so PRP can promote the proliferation of human adipose-derived stem cells [**6**].

Fractional resurfacing is a new concept in the laser field, which causes minimal disruption of the epidermis and generates macrocolumns of coagulated tissue that extend deep into the dermis. The fractional laser CO₂ treatment causes tissue tightening and collagen remodeling both initially and for a 3 to 6 months period after treatment [**7**].

Based on the results we obtained by combining fat graft with lasers and PRP in cervico-facial rejuvenation [**8**], it came up with the idea to use this association in scar treatment. 

## Methods and Results

Between 2008 and 2013, 64 patients were enrolled in the study, 21 with contractile scars and 43 with atrophic scars. There were 57 females and 7 males, age range from 18 to 46 years old. All the patients gave an informed consent, their full medical history and were photographed before and after the treatment. Their skin type ranged between III and IV on Fitzpatrick scale.

The procedure for the fat harvesting and lipofilling was as it follows:

• marking the donor and treatment sites

• harvesting fat from the flanks or the abdomen with a 10, 20 or 60 cc syringe attached to a 14 or 16 G needle or 12-hole Khouri 12 G cannula

• allow the fat to stand for a moment, discard the lower fraction (water and blood) followed by gentle centrifugation (200 rpm, 5 minutes)

• perform fat transplantation to the marked areas with a 1 cc syringe and a Fischer cannula Ø1.2–1.4, placing small droplets of fat in the tissue following Coleman’s Lipostructure technique [**9**].

For resurfacing, we used a fractional carbon dioxide (CO₂) (λ=10,600) (MedArt 610 FRx, ASAH MEDICIO A/S, Valseholmen 11-13, Denmark) at the following parameters: power 9-12 W, time 4 ms, medium density. The laser parameters were matched with individual Fitzpatrick type.

For an activated platelet rich plasma we used a standard PRP kit GLOFIN (Salo, Finnland) – from 8.5cc patient’ blood up to 2cc PRP it was obtained after 2 centrifugations, and activated with calcium chloride. They were injected in the mid and deep dermis. During the initial burst of activity within the first hour, about 95% of the presynthesized growth factors were released, and during the remaining 7 days of their viability, the platelets were synthesized together with the secret additional growth factors.

The procedure was generally performed under local anesthesia and IV sedation (70%) and orotracheal intubation (OTI) anesthesia (30%). All the patients followed a pre- and post- operating protocol. The pre-operating process consisted of blood tests (biochemistry and coagulation), anti-bruising and antiviral medication. The postoperative process consisted of antibiotic and non-steroidal anti-inflammatory drug therapy and compressive dressings for the harvesting place (24-48 hours).

The patients were hospitalized for 1 day, at departure they were recommended emollient creams and creams with SPF 50ⁱ for 1 month. The follow-up took place after 1 week, 1, 3, 6 months.

Results were gathered through photographs (Canon camera in natural and artificial light) and the following aspects were assessed on a 4-point scale as excellent, good, fair, poor: scar appearance, skin condition, symptoms, edema, ecchymosis, recovery time (Table 1).

**Table 1:**
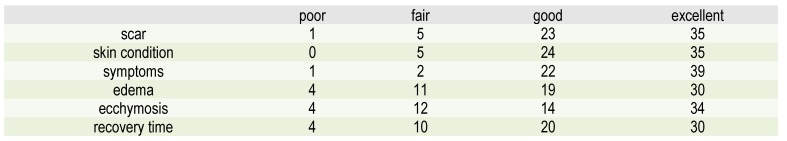
Post-treatment assessment of patient satisfaction rate at 6 months

Regarding the patients’ overall satisfaction rate, over 50% rated the treatment as excellent (55.81% in atrophic scars and 52.38% in contractile scars). Although antiviral therapy was applied, in 2 patients herpes reactivated, but it resolved in 4 days without leaving pigmented lesions.

**Fig. 1:**
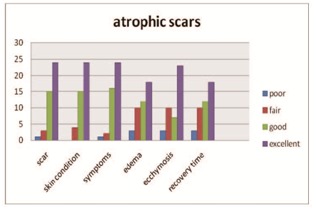
Patient evaluation in atrophic scars

**Fig. 2:**
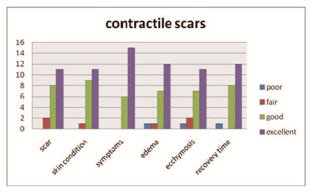
Patient evaluation in contractile scars

The most bothersome symptom was post lipofilling edema, lasting in some cases, up to 1 month, more pronounced in the atrophic scars (30.23%) compared to contractile scars (9.5%). This difference may result from the location of lesions. In the atrophic scars, acne was the main cause, so injuries were on the face, edema was more disturbing. Thus patients with atrophic lesions in several cosmetic areas (cheeks, menton and glabellas area) as well as those who experienced numerous injuries of icepick and boxcar (the riggotomies – cutting the adherence with a hypodermic needle 16 G- were more aggressive, had a greater degree of swelling and bruising (**Fig. 1,Fig. 2**).

**Fig. 3:**
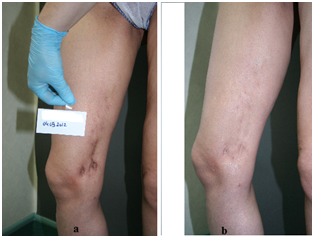
Scar post Bio Alcamide filler used on the leg (a) before (b) after treatment at 6 months

**Fig. 4:**
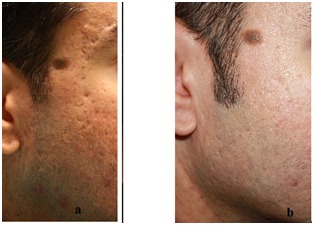
Post acne scar (a) before (b) and after treatment at 6 months

The contractile scars were found more on the abdomen and legs, so the side effects were more easily tolerated by the patient, and recovery was rapid. Most lesions were 2 years old and thus createed higher adhesions, so nearly half of the cases have been or will need a 2nd procedure (**Fig. 3,Fig. 4**).

## Discussions

Our results are matched with the results presented by Cervelli V. in 2012 [**10**], regarding the association of fat graft, PRP and laser in scar treatment. The differences were between the lasers; we used an ablative fractional laser, they used a non-ablative one, and we did all the procedure in one day, so the recovery time and side effects were more evident.

Conventional surgical lasers produce photothermal and other effects in tissue which are destructive in nature: there is a permanent change in the irradiated tissue following laser irradiation. This is true regarding the incision, vaporization and coagulation. The reaction in tissue following such laser irradiation is therefore above the survival threshold of normal tissue, causing death or disruption, classed as high level laser treatment (HLLT). Even with surgical lasers, concomitant effects leave tissue alive and well, but also stimulate the tissue, and these are classified as low level laser therapy (LLLT) [**11**].

HLLT occurs at temperatures from above 40°C and 200°C and, depending on the temperature, can achieve carbonisation, vaporization, coagulation, denaturation and degradation of proteins. In our study, the major photosurgical effects were coagulation and protein degradation, which are sufficient to induce the wound healing process.

LLLT on the other hand, works at temperatures under 40°C, or with no temperature rise at all, and induces photobioactivation without any damage. The resulting photochemical, photodynamic, photoenzymatic, and photoimmune effects act directly on cells at a subcellular organelle level to achieve a variety of results including cell repair, enhancement of cellular function and cellular proliferation. It is well accepted that the energy of photons when absorbed directly in cells or tissue during the LLLT process may affect cellular metabolism and signaling pathways. Reported results include increased cell proliferation and migration (particularly by fibroblasts), increased tissue oxygenation, modulation in the levels of cytokines, growth factors and inflammatory mediators [**12**].

The macroablative columns (MACs) associated with ablative CO₂ fractional resurfacing induce wound healing, with elastinogenesis and neocollagenesis in the upper and mid dermal layers, resulting in a much better-organized dermal matrix and younger-looking epidermis but also have an associated zone of low photon intensity surrounding the damaged tissues, thereby assisting with the acceleration of the wound healing process and also photobioactivation of the transplanted fat cells to ensure better graft take with less initial resorption. So, in this case, we can say that CO₂ laser is a non-contact LLLT device for our fat graft.

Based on the recent data released by Robert E. Marx, 2004 [**13**] and Cervelli V-2009 [**14**] on the role of platelet growth factors in accelerating the healing entitled us to assign in this technique. The immediate effect was to shorten the healing time (peeling) post laser from 7 days to 4 days and we believe that further increased the graft survival. However, further studies both in vitro and in vivo are needed to see if by mixing these factors with adipose tissue (somewhere in his processing before being reinjected) the survival of regenerative cells increases. The present study, was administered separately, due to the assumptions that blood macrophages could destroy the transplanted fat cells - Sommer B. – 2000 [**15**].

**Fig. 5:**
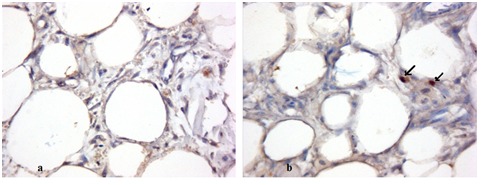
Adipocytes seen without laser stimulation (a) and with laser stimulation (b). Immunohistochemical staining forDlk1(400x) using MAb (mouse antibody) to Dlk1 (human) at dilution 1:500, shows cytoplasmic staining of preadipocytes (black arrow) in the laser and PRP-stimulated specimen at 10 days.

Given the condition that the patients were enrolled in this study, scars, obtaining histological proofs was impossible. So, we conducted another study in cooperation with our patients preparing for tummy tuck regarding histological differences between stimulated fat graft with laser and PRP and un-stimulated one (ProEstetica Medical Center Research Ethics Committee – Institutional Review Board – Resolution 7/2011). Histological examination showed a significant difference in the collagen structure, the number of adipocytes and the presence of young adipocytes between stimulated and un-stimulated fat graft (Fig. 5 ).

Given the results we can conclude that the association of an ablative laser CO2 with PRP and autologous fat graft seems to be a promising and effective therapeutic approach for atrophic and contractile scars. In general, we can affirm that the treated areas regained characteristics similar to normal skin, which are clinically objectivable, leading not only to aesthetic but also functional results. Further studies are needed to explore the potential use of this combined treatment in HIV+ patients on HAART therapy with atrophic lipodistrophy, patients with scleroderma, and patients with irradiated tissues.

**Disclosures**

None

## References

[1] Sund B (2001). New Developments in Wound Care.

[2] Zuk PA, Zhu M, Mizuno H (2001). Multilineage cells from human adipose tissue: implications for cell-based therapies. Tissue Eng.

[3] Zhu M, Zhou Z, Chen Y (2010). Supplementation of fat grafts with adipose derived regenerative cells improves long terms graft retention. Ann. Plast Surg..

[4] Colman SR, Coleman SR, Mazzolla RF (2008). Updates on structural fat grafting, Fat injection: From filling to regeneration.

[5] Pietrzak WS, Eppley BL (2005). Platelet rich plasma: biology and new technology. J Craniofac Surg.

[6] Kakuda N, Minakata T, Mitsui T (2008). Proliferation promoting effect of platelet-rich plasma on human adipose-derived stem cells and human dermal fibroblast.. Plast Reconstr Surg..

[7] Lanbach HJ, Tannous Z, Anderson RR (2006). Skin responses to fractional photothermolysis.. Lasers Surg Med..

[8] Jianu DM, Filipescu M, Jianu SA (2012). The sinergy between lasers and adipose surgery in face an neck rejuvenation: A new approach from a personal experience.. Laser Therapy..

[9] Coleman SR (1997). Facial recontouring with lipostructure.. ClinPlast Surg..

[10] Cervelli V, Nicoli F, Spallone D (2012). reatment of traumatic scars using fat grafts mixed with platelet-rich plasma, and resurfacing of skin with the 1540 nm nonablative laser.. Clinical & Experimental Dermatology..

[11] Ohshiro T (1991). Low Level LASER Therapy : Practical Aplications.

[12] Ohshiro T (2011). New classification for single-system light treatment.. Laser Therapy..

[13] Marx RE (2004). Platelet rich plasma: evidence to support its use.. J. Oral Maxilofac. Surg..

[14] Cervelli V, Gentile P, Scioli MG (2009). Application of platelet rich plasma in plastic surgery: clinical and in vitro evaluation.. Tissue Eng Part C Methods..

[15] Sommer B, Sattler G (2000). Current concepts of fat graft survival: histology of aspirated adipose tissue and review of the literature.. Dermatol Surg..

